# TWEAK Regulates Muscle Functions in a Mouse Model of RNA Toxicity

**DOI:** 10.1371/journal.pone.0150192

**Published:** 2016-02-22

**Authors:** Ramesh S. Yadava, Erin P. Foff, Qing Yu, Jordan T. Gladman, Timothy S. Zheng, Mani S. Mahadevan

**Affiliations:** 1 Department of Pathology, University of Virginia, Charlottesville, VA, United States of America; 2 Department of Neurology, University of Virginia, Charlottesville, VA, United States of America; 3 Department of Immunology, Biogen Idec, Cambridge, MA, United States of America; University of Texas MD Anderson Cancer Center, UNITED STATES

## Abstract

Myotonic dystrophy type 1 (DM1), the most common form of muscular dystrophy in adults, is caused by toxic RNAs produced from the mutant DM protein kinase (DMPK) gene. DM1 is characterized by progressive muscle wasting and weakness. Therapeutic strategies have mainly focused on targeting the toxic RNA. Previously, we found that fibroblast growth factor-inducible 14 (Fn14), the receptor for TWEAK, is induced in skeletal muscles and hearts of mouse models of RNA toxicity and that blocking TWEAK/Fn14 signaling improves muscle function and histology. Here, we studied the effect of *Tweak* deficiency in a RNA toxicity mouse model. The genetic deletion of *Tweak* in these mice significantly reduced muscle damage and improved muscle function. In contrast, administration of TWEAK in the RNA toxicity mice impaired functional outcomes and worsened muscle histopathology. These studies show that signaling via TWEAK is deleterious to muscle in RNA toxicity and support the demonstrated utility of anti-TWEAK therapeutics.

## Introduction

Myotonic dystrophy type 1 (DM1), the most common form of muscular dystrophy in adults and children, is characterized by progressive muscle wasting and weakness. DM1 is caused by a (CTG)_n_ expansion in the 3’ untranslated region (3’ UTR) of the DMPK gene [1]. The RNA expressed from the expanded allele accumulates in the nucleus and is toxic to cells [2–4]. The expanded RNA alters activity of RNA binding proteins that are splicing regulators, leading to missplicing of many downstream target genes and some of the observed phenotypes in DM1[5, 6]. Although it is well established that the repeat expansion is responsible for the muscle damage in DM1, the downstream pathways by which it causes muscle wasting is not clear. Some studies suggest that missplicing events in the target genes (e.g. *Cav1*.*1* and *BIN1*) may play a role in skeletal muscle pathology [7, 8]. Recently, we discovered that the fibroblast growth factor inducible14 (Fn14, Tnfrsf12a) receptor is significantly induced in skeletal and cardiac muscles of DM1 patients and mouse models of the disease [9]. We found that the expression of Fn14 expression correlates with severity of muscle pathology and genetic deletion of Fn14 results in improved muscle functions [9].

TWEAK (TNF-like weak inducer of apoptosis), the ligand for Fn14, is a member of the TNF superfamily of cytokines and is expressed in many tissues [10]. TWEAK has been shown to affect several biological responses including cell proliferation, differentiation, angiogenesis, apoptosis, inflammation, and fibrosis [11]. However, mice deficient in TWEAK do not exhibit overt phenotypes and are viable and healthy [12, 13]. In contrast, over-expression of TWEAK affects myoblast differentiation and muscle wasting [14–16]. There is also significant evidence to suggest that increased levels of TWEAK have adverse effects on muscle regeneration, autophagy, inflammation, and muscle metabolism [17–21]. Recently, TWEAK has been shown to modulate muscle atrophy in an amyotrophic lateral sclerosis (ALS) model [22]. TWEAK mediates it effects through binding to the Fn14 receptor (in a one ligand: one receptor fashion) [23, 24]. Since deletion of Fn14 is beneficial in a mouse model of RNA toxicity, the aim of this study was to evaluate TWEAK’s contribution to the pathology of RNA toxicity mice. Here, we find that TWEAK plays a deleterious role in DM1 pathology and that genetic deletion of *Tweak* in the mice with RNA toxicity results in increased survival, reduced muscle pathology and improved muscle function.

## Materials and Methods

### Transgenic mice

We used a mouse model of RNA toxicity (DM5) in which the *DMPK* 3’UTR with (CTG)_5_ is expressed as part of an eGFP transcript. Details about the DM5 mice are described elsewhere [3]. *Tweak*^*-/-*^ mice were obtained from Biogen Idec [12]. All experiments using the DM5/*Tweak* ^*-/-*^ mice were done with isogenic controls (DM5*/Tweak*^*+/+*^*)*. Mice were two months old. DM5/*Tweak* ^*-/-*^ and DM5*/Tweak*^*+/+*^ mice were induced with 0.2% doxycycline in the drinking water (referred to as DM5/*Tweak* ^*-/-*^ D+ and DM5*/Tweak*^*+/+*^ D+) to study the role of TWEAK in the presence of RNA toxicity. DM5/*Tweak* ^*-/-*^ and DM5*/Tweak*^*+/+*^ mice which were not induced are referred to as DM5/*Tweak* ^*-/-*^ D- and DM5*/Tweak*^*+/+*^ D-.

### Administration of soluble TWEAK in mice

To study the effects of TWEAK in vivo, DM5 mice at 2 months of age were phenotyped and then induced with 0.2% doxycycline. Subsequently, they were injected intraperitoneally (i.p.) with soluble TWEAK (30 μg/g) or an isotype control (P1.17) (30 μg/g) every 3 days for a total of two weeks. These mice were phenotyped by ECG, EMG, distance run on a treadmill and for grip strength at 1 and 2 weeks after induction of RNA toxicity as previously described [25]. At the end of 2 weeks, tissues were collected and analyzed.

### Survival study and phenotypic analysis

Twenty one mice (age two months) per study group (DM5/*Tweak*^*+/+*^ and DM5/*Tweak*^*-/-*^) were induced with 0.2% doxycycline. Induced DM5 mice typically develop phenotypes within three to five days and many die within three weeks typically from cardiac dysfunction [3, 26]. Protocols for treadmill running and grip strength, EMGs and ECGs are described elsewhere [25]. All protocols were approved by institutional committees for animal care and use. All mice were assessed pre-induction (baseline). All surviving mice were re-assessed at seven and fourteen day’s post-induction of RNA toxicity. All results were reported as retained function with reference to baseline for each mouse.

### Histology and muscle fiber size determination

H&E staining was done on serial cryostat sections (6μm) of skeletal muscles (paraspinal or quadriceps femoris) according to standard procedures and examined under a light microscope. Microscopy was performed using an Olympus IX 50 inverted microscope with fluorescent attachments and images were captured with a CCD camera. 200 X images of H&E stained skeletal muscle were used for muscle fiber size determination by measuring cross-sectional area of each muscle fiber. At least five mice per group were analyzed, and for each mouse at least three different images and at least 300 fibers were analyzed. Muscle fiber size was measured using AxioVision^™^ V4.8.2.0 (Carl Zeiss MicroImaging).

### Immunofluorescence and western blot analysis

Protocols for immunofluorescence and western blotting for NF-κB2 are described elsewhere[9]. Immunofluorescence for NF-κB2 was performed using anti NF-κB2- p52 (sc-298, Santa Cruz Biotech, Inc). The following antibodies were used for western blot analysis: NF-κB2 (p100 and p52; Cell Signaling Tech^®^ #4882), and GAPDH (Ambion^®^ #4300). Blots were scanned and relative protein expression was quantified using Image Quant5.1^™^.

### RNA isolation and quantitative RT-PCR assays

Total RNA was extracted according to previously described protocols [27]. RNAs (1 μg) were treated with DNase I (Ambion^®^, cat.# 1907) and then cDNA was synthesized using the QuantiTect^™^ Reverse Transcription Kit (Qiagen^®^, Cat. No. #20531). qRT-PCR was done using the BioRad iCycler^™^ and detected with SYBRGreen^™^ dye. All assays were done in duplicate, and the data was normalized to an endogenous control (*Gapdh*). The values were subjected to a 2^(-ΔΔCt)^ formula to calculate the fold change between the control and experimental groups. Fold change post-induction was calculated relative to appropriate un-induced genetically identical mice. For primer sequences, PCR conditions and PCR efficiencies see S1 Table. We calculated PCR efficiency from the slope of the standard curve using the following formula: E = (10^(-1/slope)-1)*100. All the real-time PCR assays were optimized to ensure efficiency between 90–105%.

### Human skeletal muscle samples

cDNAs from tibialis anterior muscle biopsies were provided by Dr. Charles A Thornton for the analysis of *TWEAK* mRNA levels in human skeletal muscle samples [9]. These subjects were recruited as part of an IRB approved protocol as part of studies supported by National Institute of Health grants (U54NS48843, UL1RR024160).

### Statistical Analysis

All experiments were performed with at least 5 mice per group. Mortality data were analyzed using a Breslow test. Data sets were first analyzed for outliers using the Grubb’s test. For real-time PCR, outliers were assessed prior to calculation of fold change. Once outliers were removed, the data set was analyzed for normality. If normal, two-tailed student’s T-tests were employed to assess significance, with attention paid to equal versus unequal variance. Data were expressed as either mean ± SD or mean ± SEM. P<0.05 was considered statistical significant unless otherwise specified.

### Study Approvals

All animal protocols were approved by the institutional ICAUC at the University of Virginia.

## Results

### TWEAK deficiency is beneficial in RNA toxicity

We have a well characterized inducible/reversible mouse model of RNA toxicity (referred to as DM5) in which expression of the toxic RNA is inducible by administering 0.2% doxycycline. This results in many features of DM1 including myotonia, abnormal muscle pathology, and RNA splicing defects [3]. Using this mouse model, we have previously demonstrated that Fn14 signaling plays an important role in mediating muscle damage [9]. However, the role of its ligand, TWEAK, has not been as fully delineated. To clearly evaluate the role of TWEAK in RNA toxicity, *Tweak* knockout mice were bred with DM5 homozygotes to obtain homozygous DM5/*Tweak* ^*-/-*^ mice, as well as a control group of DM5/*Tweak*^*+/+*^ mice in the appropriate mixed genetic background. As reported previously by other groups [13, 28], *Tweak*^*-/-*^ mice are healthy and do not develop any overt muscle specific phenotypes. We confirmed *Tweak* deficiency by real-time PCR (S1 Fig). We obtained baseline functional data by electromyography (EMG), electrocardiography (ECG), treadmill running and forelimb grip strength on all mice prior to induction of RNA toxicity and found no significant differences between the two groups (S2 Table). All surviving mice were reevaluated at one and two weeks post-induction. We confirmed that toxic RNA and *Fn14* mRNA levels were equivalent between study groups by qRT-PCR (S1 Fig). We also found no changes in *TWEAK* mRNA levels when comparing skeletal muscles from unaffected individuals and those with DM1 (S2 Fig)

**Fig 1:**
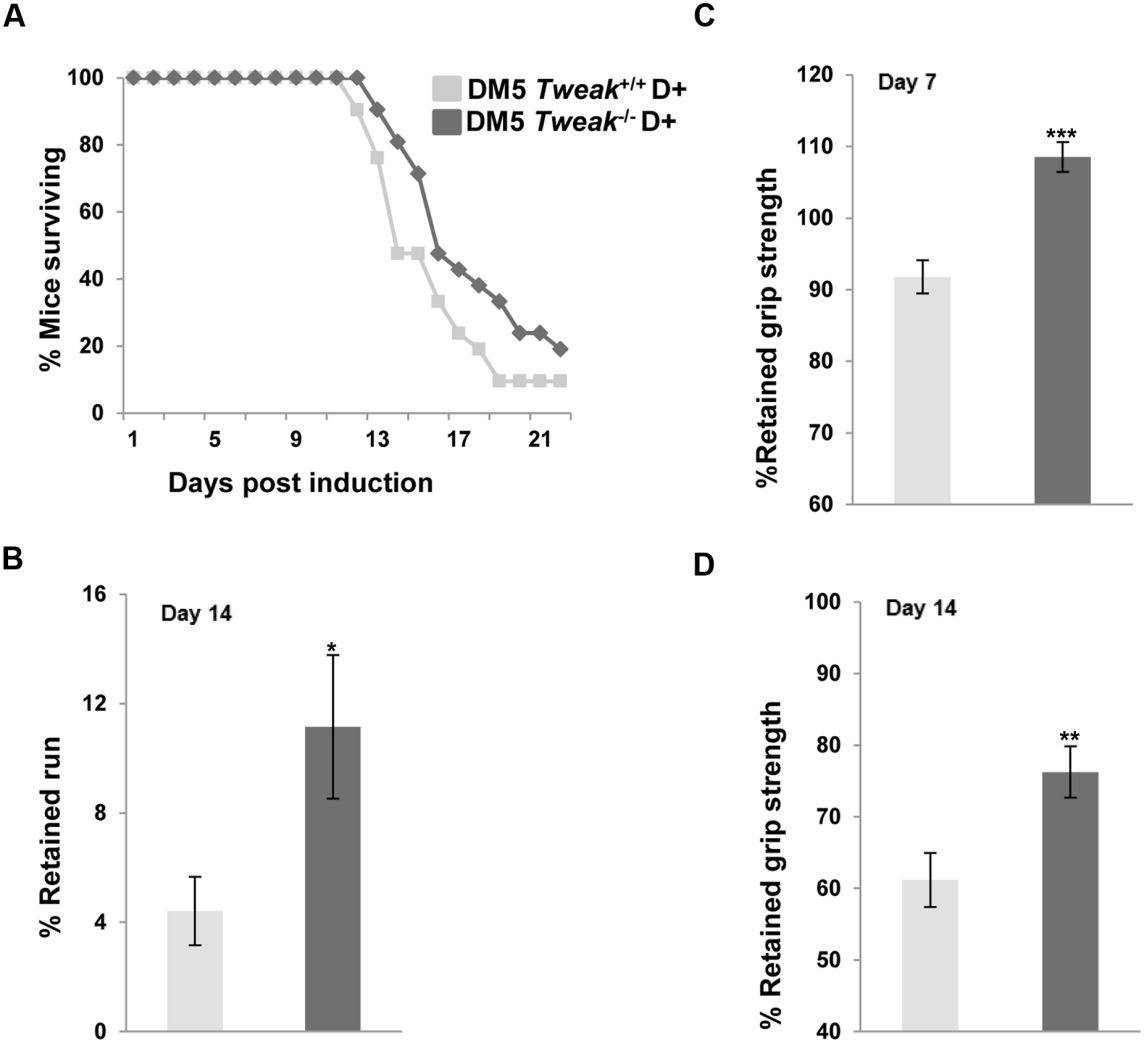
Deletion of *Tweak* leads to improved functions. (A) Survival curves showing a trend towards decreased mortality in DM5/Tweak^-/-^ D+ mice as compared to DM5/Tweak^+/+^ D+ mice (p = 0.068). N = 21 mice of two months of age were used for survival study in each group. (B) Improvements in treadmill running distances at day 14 (*p = 0.049) and (C, D) forelimb grip strength at day 7 (***p = 0.00006) and day14 (**p = 0.008) were noted in DM5 D+ mice lacking *Tweak*; (DM5/*Tweak*^+/+^ D+ (n = 12), DM5/*Tweak*^-/-^ D+ (n = 15)). Errors bars are mean ± s.e.m.

**Fig 2:**
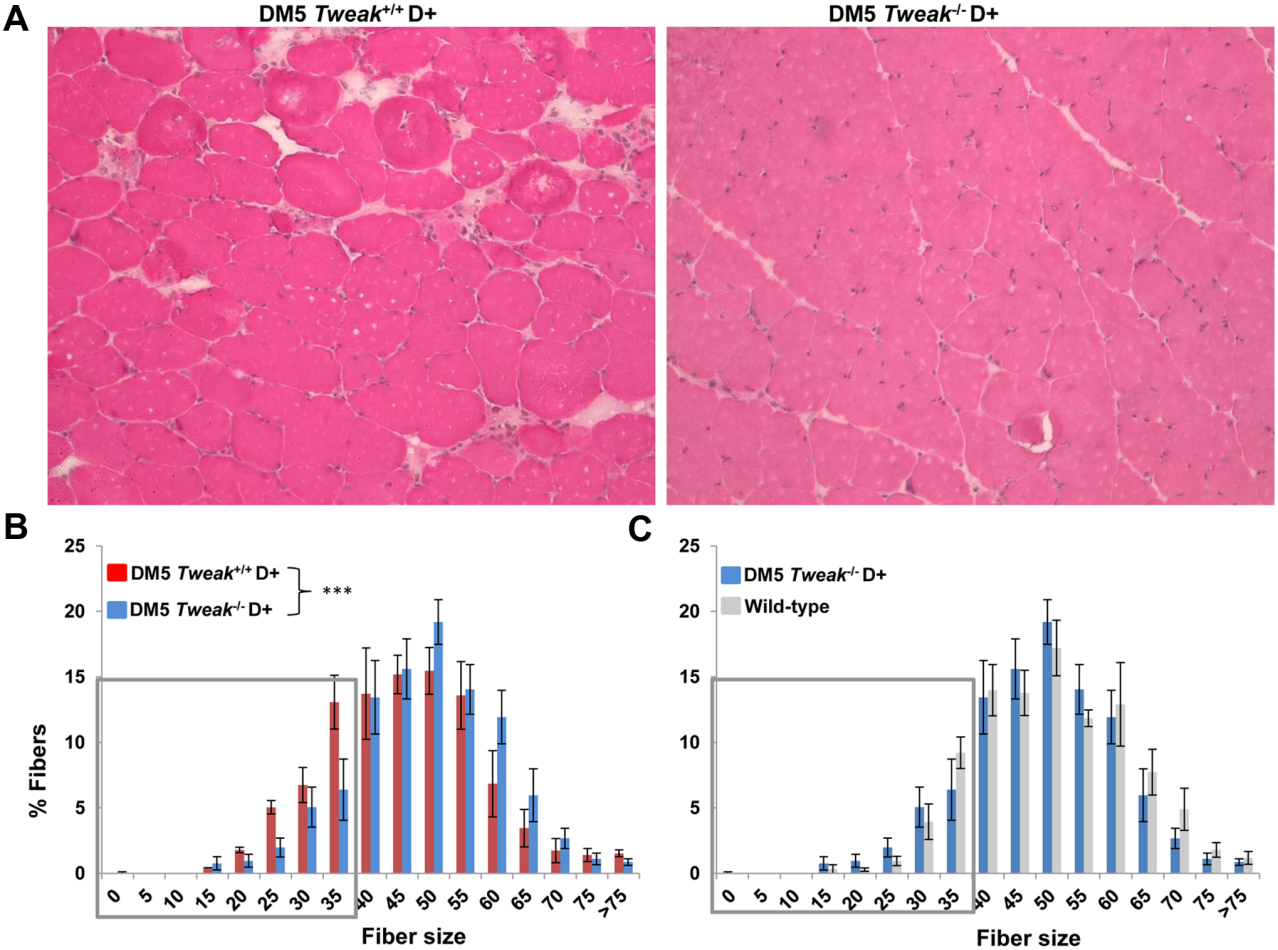
Tweak deficiency improves muscle histology. (A) H&E staining of skeletal muscle showing improved morphology in skeletal muscles obtained from DM5/*Tweak*^*-/-*^D+ mice as compared to samples from DM5/*Tweak*^+/+^ D+ mice (muscle collected at 14 days post induction of RNA toxicity). Note the decreased fiber atrophy and reduced central nuclei in the DM5/*Tweak*^*-/-*^D+ mice. (B) Histogram of skeletal muscle fiber diameter distribution shows that *Tweak* deficiency (DM5/*Tweak*^*-/-*^ D+ (n = 4)) (blue) corrects the skew towards smaller fibers caused by RNA toxicity (DM5/*Tweak*^+/+^ D+ (n = 3)) (red). At least 300 fibers were analyzed per mouse. The grey box represents fibers <35um in diameter. (***p = 0.0001). (C) Histogram of skeletal muscle fiber diameter sizing shows that *Tweak* deficiency in the RNA toxicity mice (DM5/*Tweak*^*-/-*^ D+ (n = 4)) (blue) results in a distribution similar to that seen in wildtype mice (n = 3) (grey).

To assess the effect of TWEAK on survival, DM5/*Tweak* ^*-/-*^ and DM5/*Tweak*^+/+^ mice with RNA toxicity (n = 21/group) were followed post-induction. No differences in EMG and ECG studies were observed with all tested mice developing robust myotonia and similar degrees of cardiac conduction abnormalities at two weeks post-induction. There was a trend towards a survival benefit in DM5/*Tweak* ^*-/-*^ D+ mice with 15 of 21 alive at 14 days and 7 of 21 alive at 18 days (Fig 1A) as compared to DM5/*Tweak*^+/+^ D+ mice with 10 of 21 alive at 14 days and 2 of 21 alive at 18 days (p = 0.068). The DM5/*Tweak* ^*-/-*^ D+ mice also had significantly better treadmill run fitness at day 14 (p = 0.049) (Fig 1B) and forelimb grip strength at day7 (p = 0.001), and at day 14 (p = 0.005) as compared to DM5/*Tweak*^*+/+*^D+ mice (Fig 1C and 1D). We also observed preservation of muscle architecture in the DM5/*Tweak* ^*-/-*^ D+ mice when compared to DM5/*Tweak*^*+/+*^D+ mice (Fig 2A). Muscle fiber diameter analysis also indicated a marked shift towards smaller and atrophic fibers (p = 0.0001) in the DM5/*Tweak*^*+/+*^D+ mice as compared to DM5/*Tweak* ^*-/-*^ D+ mice (Fig 2B). In fact, loss of TWEAK in the DM5/*Tweak* ^*-/-*^ D+ mice resulted in a fiber distribution that was similar to the wild-type mice (Fig 2C). These data suggest TWEAK deficiency is beneficial to muscle function and histopathology in the presence of RNA toxicity.

### TWEAK adversely affects skeletal muscles in RNA toxicity mice

**Fig 3:**
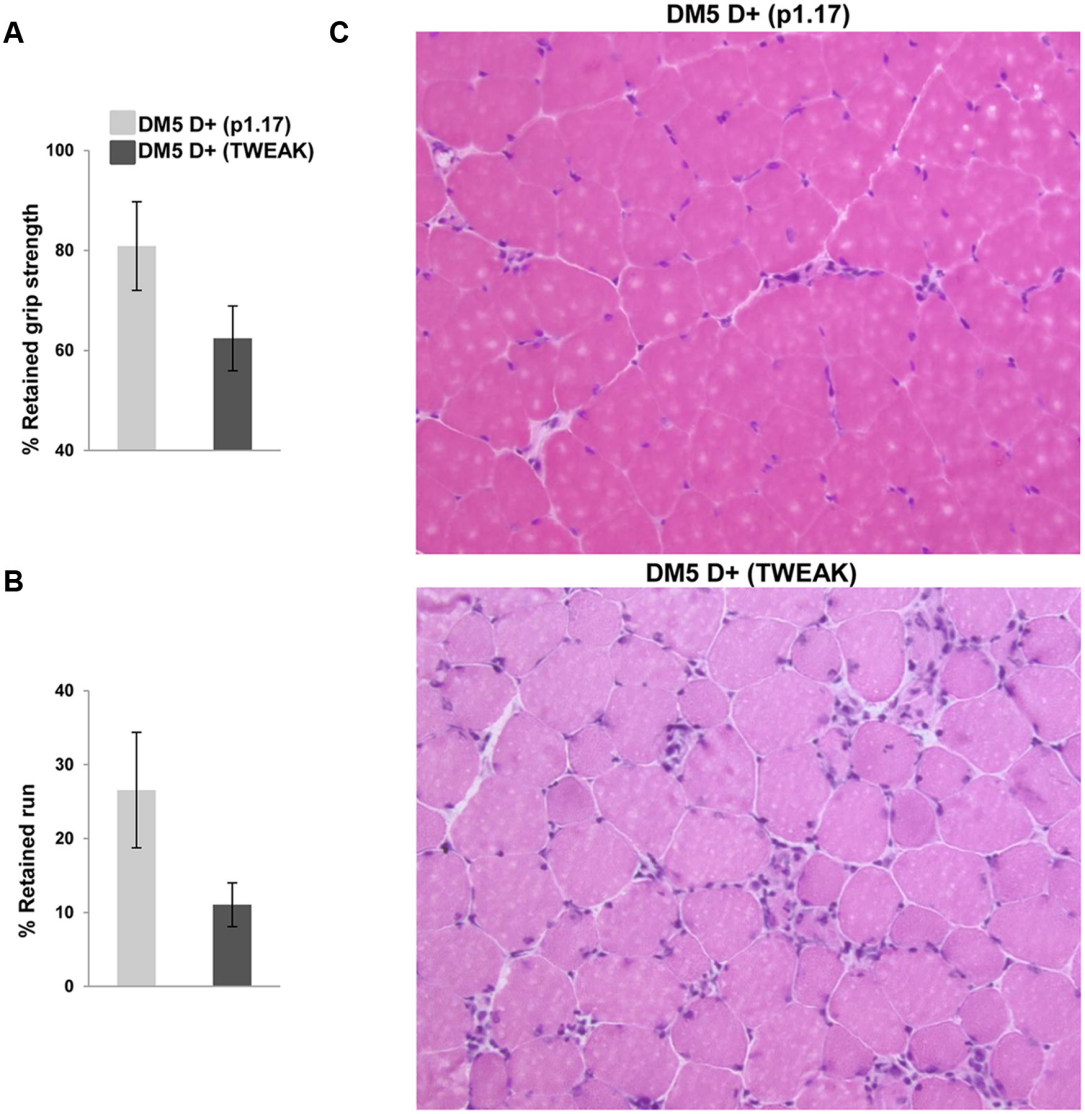
Exogenous administration of Tweak leads to impaired function and muscle histopathology. (A) Grip strength analysis at two weeks post RNA toxicity induction shows a trend towards decreased retained grip strength in DM5 D+ mice given soluble TWEAK (30 μg/g/q3d) as compared to an isotype control (P1.17) (30 μg/g/q3d); (n = 4–5 per group); errors bars are mean±s.e.m. All mice were two months of age when given doxycycline. TWEAK and p1.17 were given every three days over the course of the two week experiment. (B) Treadmill running analysis at two weeks post RNA toxicity induction shows a trend towards decreased retained run distance in DM5 D+ mice given soluble TWEAK (30 μg/g/q3d) as compared to an isotype control (P1.17) (30 μg/g/q3d); (n = 4–5 per group); errors bars are mean±s.e.m. (C) H&E staining of skeletal muscle shows increased pathology in mice treated with TWEAK. Note obvious increases in central nuclei, atrophic fibers and fiber size variation.

It has previously been shown that over-expression of TWEAK or exogenous administration of TWEAK in wildtype mice has detrimental effects on skeletal muscle [16]. To study if the increased expression of Fn14 increased the sensitivity of the RNA toxicity mice to the effects of TWEAK, DM5 mice induced with doxycycline at the age of 2 months (DM5 D+) were injected intraperitoneally (i.p.) with soluble TWEAK (30 μg/g) or an isotype control (P1.17) (30 μg/g) every 3 days for a total of two weeks. The mice were phenotyped by ECG, EMG, treadmill run, and grip strength, prior to induction and at 1 and 2 weeks after induction of RNA toxicity. There were no differences noted between the two groups prior to RNA toxicity induction. After induction, both groups of mice developed similar levels of robust myotonia and cardiac conduction defects. However, the TWEAK administered mice showed a trend towards decreased forelimb grip strength (p = 0.15) and treadmill run fitness (p = 0.11) at 2 weeks (Fig 3A and 3B). At the end of 2 weeks, mice were sacrificed and skeletal muscles were collected. H&E analyses of muscle sections clearly showed increased numbers of central nuclei and atrophic fibers in TWEAK-administered mice as compared with the isotype treated controls (Fig 3C). These data suggest that the presence of TWEAK plays a deleterious role in muscle function and contributes to abnormal histopathology in the presence of RNA toxicity.

### TWEAK modulates the expression of downstream target genes

**Fig 4:**
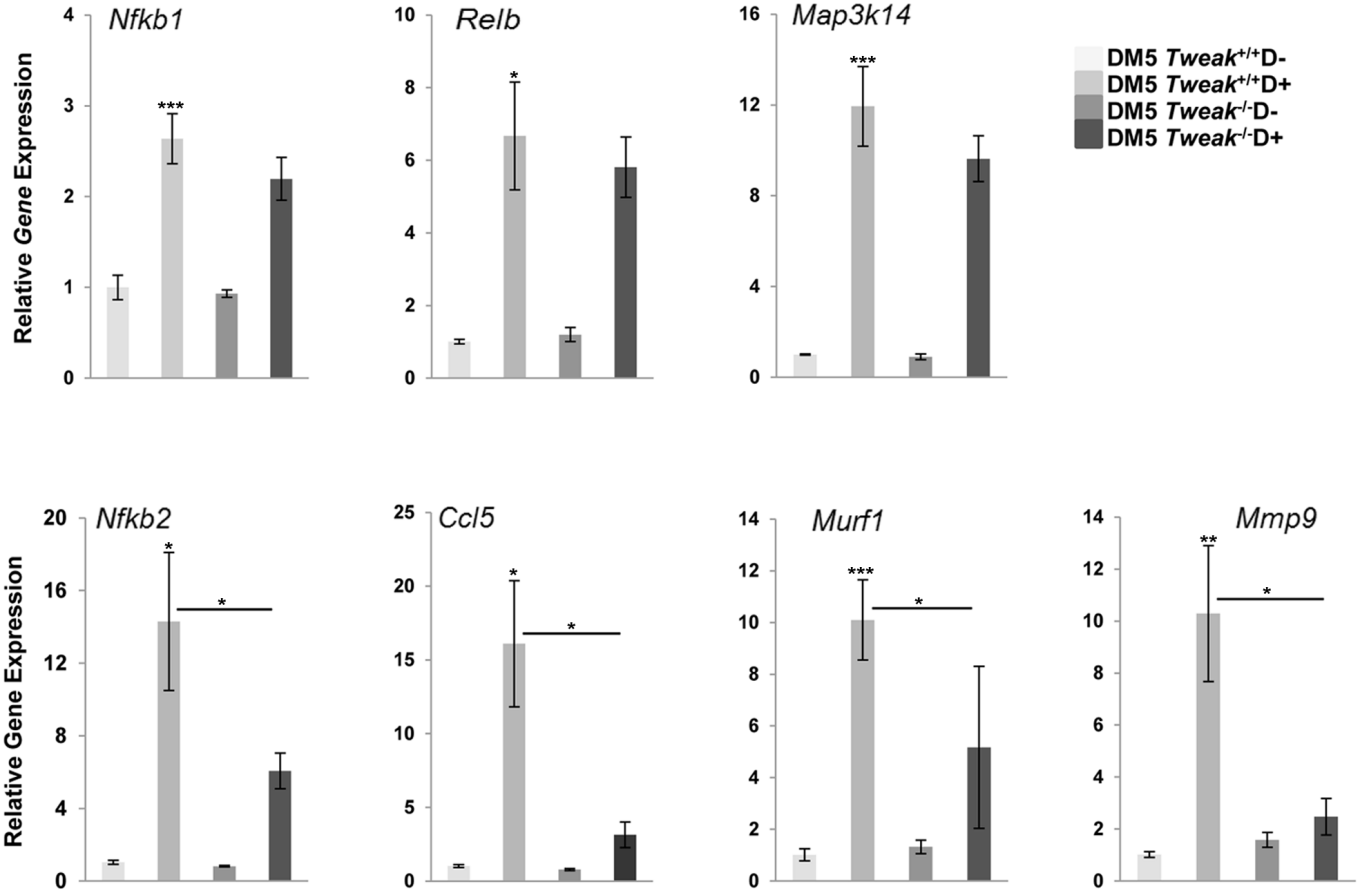
TWEAK regulates the expression of NF-κB genes and downstream targets in mice with RNA toxicity. Quantitative RT-PCR shows RNA toxicity (DM5/*Tweak*^+/+^ D+) increased the expression of NF-κB genes (*Nfkb1*, *RelB*, *Map3k14*, *Nfkb2)* and downstream target genes (*Ccl5*, *Murf1*, *Mmp9*). TWEAK deficiency in DM5 D+ mice (DM5/*Tweak* ^-/-^ D+) significantly reduced expression of *Nfkb2*, *Ccl5*, *Murf1*, *and MMP9*. (n≥5/group); error bars are mean±s.e.m.; *p<0.05, **p<0.01, ***p<0.001 (Student’s *t* test).

**Fig 5:**
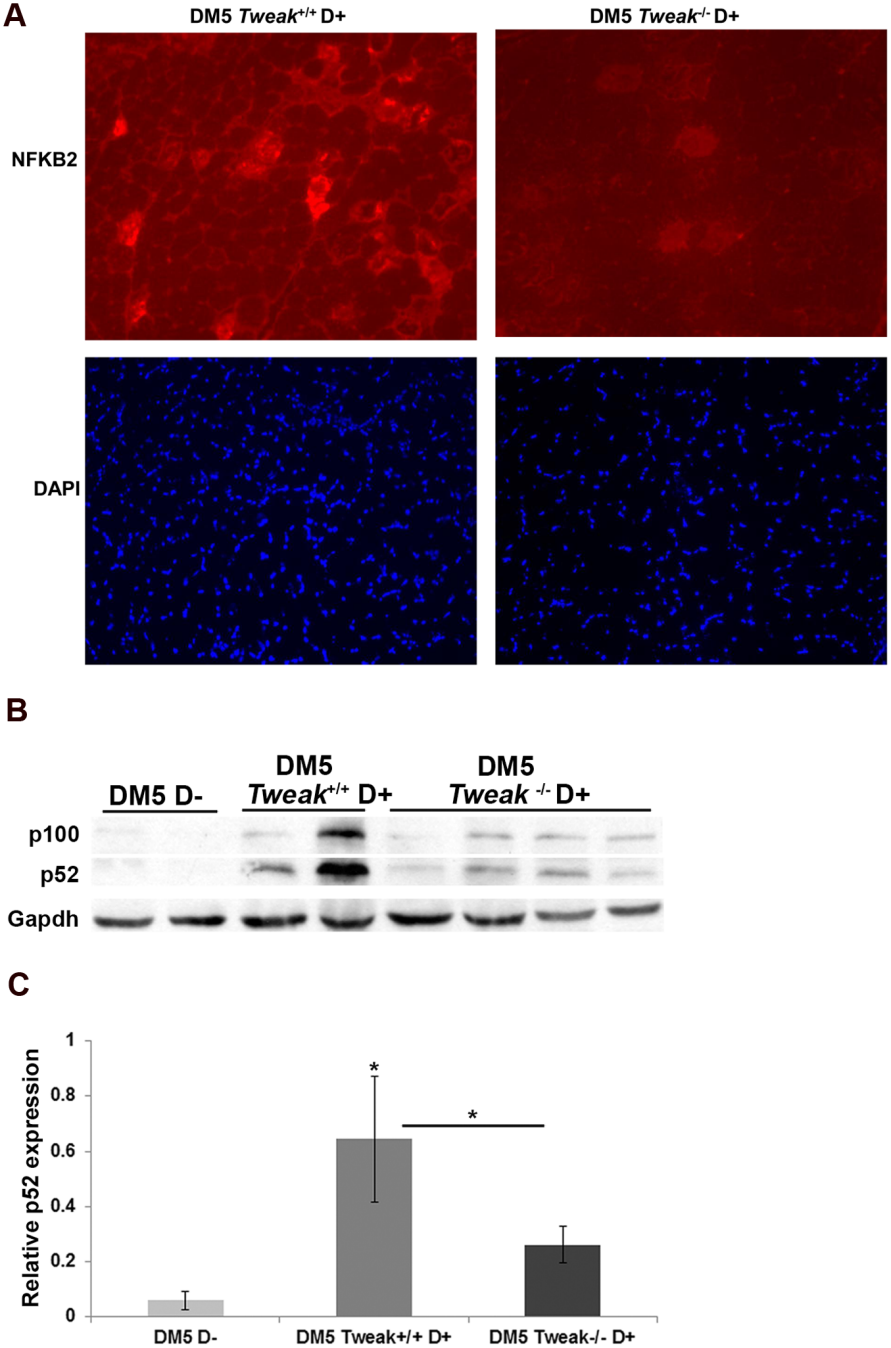
Deficiencies of TWEAK attenuate NF-ĸB2 activation in the RNA toxicity mice. (A) Immunofluorescence shows decreased NF-ĸB2 in skeletal muscle from DM5/*Tweak*^*-/-*^ D+ mice as compared to DM5/*Tweak*^*-+/+*^ D+ mice. Image acquisition settings were the same for both groups. (B) Western blot for NF-κB2 detects both the p100 isoform and the processed p52 isoform in skeletal muscle extracts; uninduced mice (DM5 D-), induced mice wildtype for *Tweak* (DM5/*Tweak*^*-+/+*^ D+), and induced mice deficient for *Tweak* (DM5/*Tweak*^*-/-*^ D+). (C) Quantification of western blots for NF-κB2 shows that p52 levels in TWEAK deficient RNA toxicity mice (DM5/*Tweak*^*-/-*^ D+) was decreased significantly as compared to RNA toxicity mice wildtype for *Tweak* (DM5/*Tweak*^*-+/+*^ D+). n = 4 per group used for analysis; error bars are mean ± s.e.m.; (*p = 0.05 (Student’s t test)).

**Fig 6:**
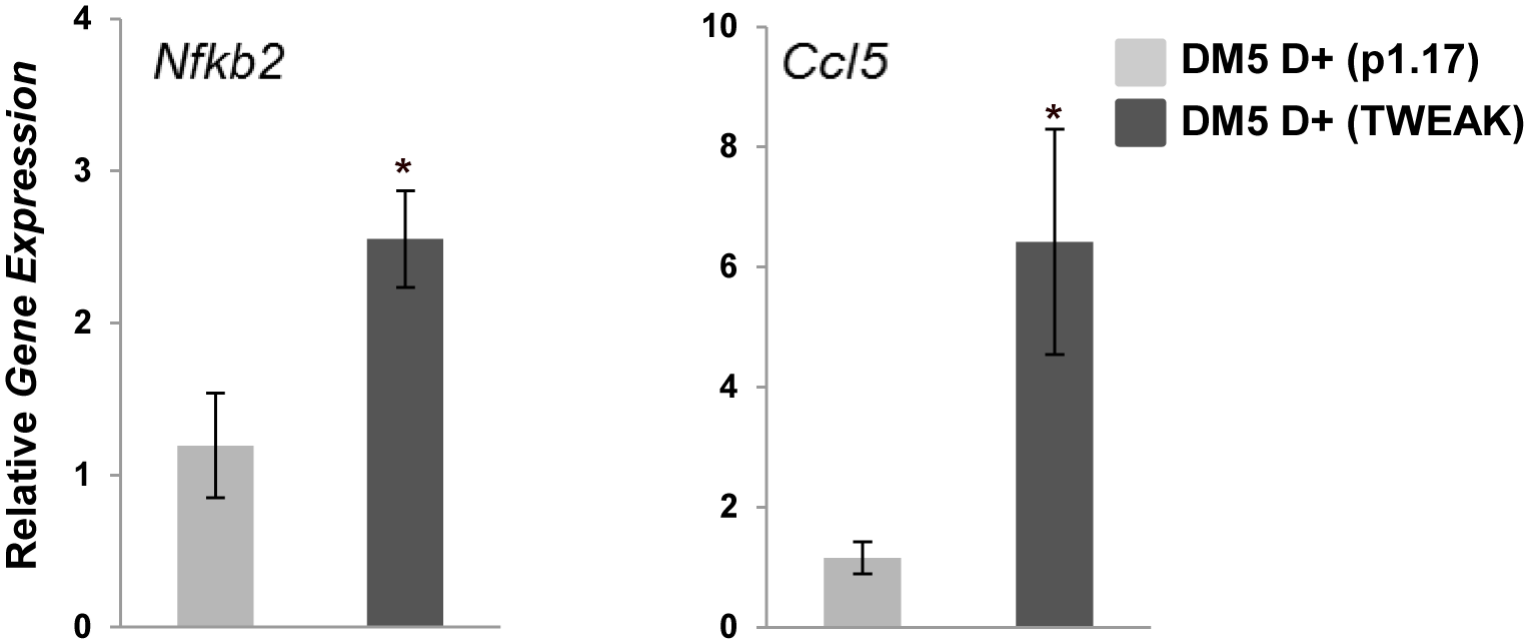
Administration of TWEAK leads to increased expression of NF-κB related genes in mice with RNA toxicity. Quantitative RT–PCR shows that systemic administration of TWEAK to DM5 D+ mice leads to significantly upregulated expression of *Nfkb2 and Ccl5* as compared to mice administered an isotype control (p1.17). (n≥5/group); error bars are mean±s.e.m.; *p<0.05(Student’s *t* test).

We’ve previously demonstrated that activation of NF-κB pathways in the RNA toxicity mice occurs partially via Fn14 [9]. To more clearly delineate the role of TWEAK in this process, we studied skeletal muscles from DM5/*Tweak* ^*-/-*^ D+ mice in comparison to DM5/*Tweak*^*+/+*^D+ mice. qRT-PCR analyses showed that the DM5/*Tweak*^*+/+*^D+ mice had significantly increased gene expression of key components of the NF-κB pathway: *Nfkb1* (p105/p50), *RelB*, *Map3k14* (NIK) and *Nfkb2* (p100/p52) (Fig 4). In addition we found higher expression of several downstream target genes (*Ccl5*, *Murf1*, and *Mmp9*) that had previously been shown to be affected by increased activity of the TWEAK/Fn14 pathway [15, 17, 29] (Fig 4). Notably, in the DM5/*Tweak* ^*-/-*^ D+ mice, we found significant attenuation of the induction of *Nfkb2* (p = 0.04) and the downstream target genes, *Ccl5* (p = 0.025), *Murf1* (p = 0.014), and *Mmp9* (p = 0.023) (Fig 4). The decreased expression of NF-κB2 was confirmed by immunofluorescence (Fig 5A), and western blotting was used to confirm that the increased expression of NF-κB2 and increased processing of p100-p52 observed in DM5/*Tweak*^+/+^ D+ mice were decreased by TWEAK deficiency (Fig 5B and 5C). Notably, DM5 D+ mice which were administered TWEAK also showed significant increased expression of *Nfkb2* (p = 0.02) and *Ccl5* (p = 0.024) when compared to the p1.17 isotype control treated group (Fig 6).

## Discussion

The role of TWEAK in mediating muscle damage in non-dystrophic conditions has been investigated in detail using mice [30]. TWEAK activates multiple cellular pathways including canonical and non-canonical NF-κB pathways. The activation of NF-κB pathways has been linked to skeletal muscle wasting in disease conditions [31]. Recently, we have also shown that RNA toxicity activates both the classical (canonical) and alternate (non-canonical) NF-κB pathways in mice and individuals with DM1, and that the Fn14 deficiency modulates these effects [9]. The results from the current studies reinforce the role of the TWEAK/Fn14 pathway in skeletal muscles under various disease conditions.

TWEAK and Fn14 are thought to function in a 1:1 ligand receptor fashion, with much of the control of the pathway mediated specifically through Fn14 levels [23, 24]. Typically, Fn14 is expressed at low levels in normal tissues, but upon damage, its expression is highly upregulated [24, 32]. This is thought to play a beneficial role in the short-term as part of a damage and regenerative response, but continued long-term expression of Fn14 and chronic stimulation through the TWEAK/Fn14 pathway has been suggested to play a deleterious role in disease states [32, 33]. It has also been postulated that Fn14 auto-activation can occur, leading to downstream effects even in conditions where local circulating TWEAK levels are not elevated [34]. We were interested in understanding more thoroughly, how much of a role the ligand (TWEAK) plays in RNA toxicity to better understand its therapeutic potential (given that a therapeutic already exists to target the ligand itself).

In this study, we identify TWEAK as a regulator of muscle dysfunction in the presence of RNA toxicity. Abrogating TWEAK function (using a genetic knockout) in an RNA toxicity background, led to an improvement in grip strength and run capabilities. We also showed that muscle architecture is better preserved in RNA toxicity mice lacking TWEAK when compared to TWEAK competent counterparts. Consistent with these observations, chronic administration of TWEAK led to impaired muscle function and reproducible muscle histopathology. Previously, we showed that mice with RNA toxicity demonstrate a TWEAK/FN14 dependent activation in NF-κB [9]. Furthermore, in these mice, we showed the beneficial effects of Fn14 deficiency with respect to muscle regeneration, autophagy, inflammation and muscle metabolism and activation of the NF-κB pathways. Here, we report a similar attenuation of the NF-κB response in the absence of TWEAK that correlated with improved histologic and functional outcomes in the RNA toxicity mice. This study shows a clear effect of TWEAK on damage mediated by RNA toxicity, though the effect of Fn14 over-expression does appear more significant. This is not surprising given that high levels of Fn14 have been reported to result in some degree of ligand independent activation of the TWEAK/Fn14 pathway. However, the fact that we did see beneficial effects strongly supports our previous observations that targeting TWEAK represents a viable therapeutic strategy in DM1 associated RNA toxicity.

## Conclusions

Our data demonstrate that the genetic deletion of TWEAK is beneficial in RNA toxicity mice. Consistent with the genetic experiments, administration of exogenous TWEAK led to impairment in muscle histology and muscle function. This adds significant support to the proposition that blocking this pathway could modify or slow progression of muscle disease and loss, and improve the quality of life for DM1 patients.

## Supporting Information

S1 Fig Characterization of Tweak knock-out mice. (A) Quantitative RT-PCR shows no expression of *Tweak* mRNA in DM5/*Tweak*^-/-^ D+ mice. (B, C) Quantitative RT-PCR shows no change in the expression of toxic RNA (GFP) and *Fn14* mRNA in DM5/*Tweak*^-/-^ D+ mice as compared to DM5/*Tweak*^+/+^ D+ mice.
(TIF)

S2 Fig TWEAK levels are unchanged in DM1. Quantitative RT-PCR shows no significance differences in *TWEAK* mRNA in human skeletal muscle tissues from normal individuals and individuals with DM1 (n = 11 for normal and n = 19 for DM1 patients).
(TIF)

S1 Table Real-time PCR assay primers and conditions. (DOCX)

S2 Table Phenotypic analysis of DM5/*Tweak*^-/-^ and DM5/*Tweak*^+/+^ un-induced mice. (DOCX)
