# Correction: TWEAK Regulates Muscle Functions in a Mouse Model of RNA Toxicity

**DOI:** 10.1371/journal.pone.0192816

**Published:** 2018-02-08

**Authors:** The PLOS ONE Staff

The following information is missing from the Funding section: This study was funded through grants obtained from: National Institutes of Arthritis, Musculoskeletal and Skin Diseases (http://www.niams.nih.gov/). Grants: R01AR045992 (MSM), R01AR062189 (MSM), R01AR052771 (MSM).

The publisher apologizes for the error.

## References

[1] Yadava RS, Foff EP, Yu Q, Gladman JT, Zheng TS, Mahadevan MS (2016) TWEAK Regulates Muscle Functions in a Mouse Model of RNA Toxicity. PLoS ONE 11(2): e0150192 https://doi.org/10.1371/journal.pone.0150192 26901467 10.1371/journal.pone.0150192 PMC4762946

