# Supplementary figures and images for: TWEAK Regulates Muscle Functions in a Mouse Model of RNA Toxicity
Source: PLoS One. 2016 Feb 22;11(2):e0150192. doi: 10.1371/journal.pone.0150192 (PMC4762946; doi:10.1371/journal.pone.0150192)

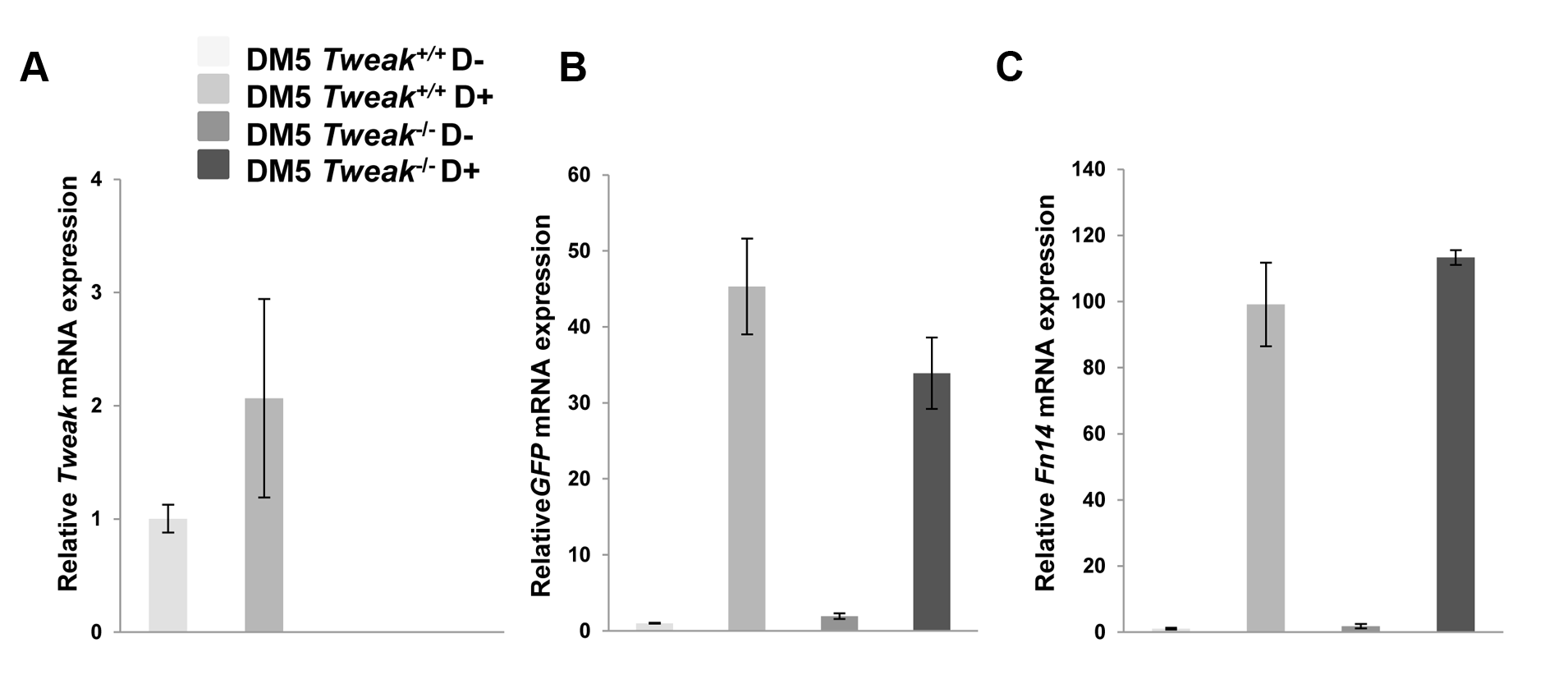

Supplement: S1 Fig — (A) Quantitative RT-PCR shows no expression of Tweak mRNA in DM5/Tweak-/- D+ mice. (B, C) Quantitative RT-PCR shows no change in the expression of toxic RNA (GFP) and Fn14 mRNA in DM5/Tweak-/- D+ mice as compared to DM5/Tweak+/+ D+ mice. (TIF) [file pone.0150192.s001.tif]

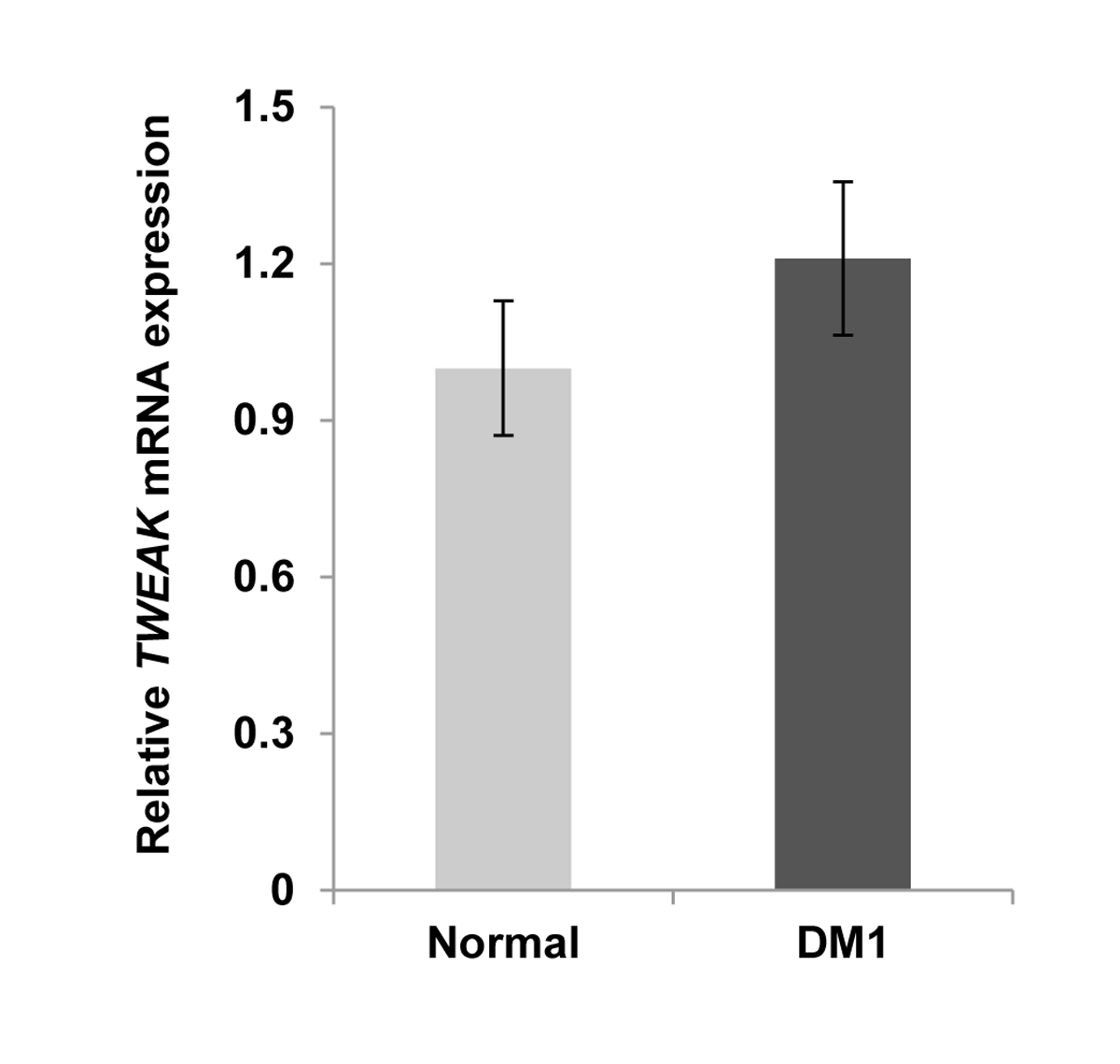

Supplement: S2 Fig — Quantitative RT-PCR shows no significance differences in TWEAK mRNA in human skeletal muscle tissues from normal individuals and individuals with DM1 (n = 11 for normal and n = 19 for DM1 patients). (TIF) [file pone.0150192.s002.tif]
