# Supplementary material for: TWEAK Regulates Muscle Functions in a Mouse Model of RNA Toxicity
Source: PLoS One. 2016 Feb 22;11(2):e0150192. doi: 10.1371/journal.pone.0150192 (PMC4762946; doi:10.1371/journal.pone.0150192)
Supplement: S1 Table — (DOCX) [file pone.0150192.s003.docx]

**S1 Table.** **Real-time PCR assay primers and conditions.**

| **Gene** | **Forward primer** | | **Reverse primer** | **Annealing**  **Temp.**  **(°C)** | **PCR**  **efficiency (%)** | |
| --- | --- | --- | --- | --- | --- | --- |
| *Fn14* | 5’-AGGCTACTGTGGCCCATTCTG-3’ | | 5’-CCCTCTCCACCAGTCTCCTCTA-3’ | 62 | 94 | |
| *Tweak* | 5’- TGCCTTGGCCTCCTGCTGGTCGT-3’ | | 5’-GCCGGACTAGTTGTTCCAAGAAA-3’ | 65 | 102 | |
| *Gapdh* | 5’-AGGTCGGTGTGAACGGATTTG-3’ | | 5’- TGTAGACCATGTAGTTGAGGTCA-3’ | 62 | 93 | |
| *Nfkb2* | 5’-CCAGCCCATCCATGACAGCA-3’ | | 5’-GGAACACAATGGCATACTGTT-3’ | 57 | 101 | |
| *Nfkb1* | 5’-GACCTGAGCCTTCTGGGACC-3’ | | 5’- CATGGCAGGCTATTGCTCATC-3’ | 59 | 104 | |
| *RelB* | 5’-AGGATCTGCTTCCAGGCCTC-3’ | | 5’- ATTCGGCAAATCCGCAGCTCT-3’ | 63 | 97 | |
| *Map3k14* | 5’-GAGGCCGTGGAGAAGAGCC | | 5’- GCATGGGCCACATTGTTGGG-3’ | 61.4 | 103 | |
| *Ccl5* | 5’-CTGCAGCTGCCCTCACCATCATCCT | | CTAGCTCATCTCCAAATAGTTGATGTATTCTTGAACC | 62 | 98 | |
| *Murf1* | | 5’-TAACTGCATCTCCATGCTGGTG-3’ | 5’-TGGCGTAGAGGGTGTCAAACTT-3’ | 60 | | 101 |
| *Mmp9* | | 5’-GCGTGTCTGGAGATTCGACTTG-3’ | 5’-CATGGTCCACCTTGTTCACCTC-3’ | 61.4 | | 99 |
| *TWEAK* | | 5’-GTGGTCAGTTTGGGGAGCCGG-3’ | 5’-CCGAACTAGTCGGTTCAGGAAA-3’ | 62 | | 96 |
| *GAPDH* | | 5’-GAAGGTGAAGGTCGGAGTC-3’ | 5’-GAAGATGGTGATGGGATTTC-3’ | 60 | | 105 |
