# Supplementary material for: TWEAK Regulates Muscle Functions in a Mouse Model of RNA Toxicity
Source: PLoS One. 2016 Feb 22;11(2):e0150192. doi: 10.1371/journal.pone.0150192 (PMC4762946; doi:10.1371/journal.pone.0150192)
Supplement: S2 Table — (DOCX) [file pone.0150192.s004.docx]

**S2 Table.** **Phenotypic analysis of DM5/*Tweak*^-/-^ and DM5/*Tweak*^+/+^ un-induced mice.**

| **Phenotypes** | **DM5/*Tweak*^-/-^**  **(n=7)** | **DM5/*Tweak*^+/+^**  **(n=11)** | **ttest** |
| --- | --- | --- | --- |
| Run distance | 504±136 | 596±159 | 0.22 |
| Grip strength | 158±14 | 163±14 | 0.68 |
| EMG | 0 | 0 | - |
| ECG | 0.035±0.0043 | 0.036±0.0034 | 0.47 |
